# Bacteriology in the Diagnosis of Diphtheria

**Published:** 1894-08-18

**Authors:** 


### Bacteriology in the Diagnosis of Diphtheria.

Among the more interesting papers read before the Health Congress, recently held in London, was a communication by Dr. Hermann Biggs, Chief Inspector of Pathology to the Board of Health of the City of New York, describing the method there adopted for the prompt diagnosis of diphtheria.

Recent bacterial investigations, he says, have shown that a considerable proportion of the cases of exudative inflammation of the throat and upper air passages, commonly considered as diphtheria, and having the anatomical appearances found in that disease, are not true diphtheria. It has also been shown that a considerable number of cases presenting symptoms by no means characteristic of true diphtheria are found on bacteriological examination to be examples of that disease, and that while in true diphtheria, as so defined, the mortality is very high and the danger of transmission to others is great, in false diphtheria the mortality is low and the danger of infection slight. On making a bacteriological examination of the cases in the hospitals of New York, cases sent in as diphtheria, and accepted by the physicians as such, he found that 40 per cent. of them were not true diphtheria. The difference between the cases of true diphtheria, in which the bacillus was found, and the false diphtheria, in which it was not discoverable, in regard to mortality was very striking. The latter, however severe they might seem—and some of them were very bad cases indeed—being less than 2 per cent., and in cases above five years old practically nil; while in 4,000 cases in which the bacillus was discovered, mild as many of them appeared at the time of investigation, the mortality averaged 30 per cent. Such confidence have the authorities in New York in their method of diagnosis that these non-bacillary cases are now excluded from the hospitals. On the other hand, it is maintained that true diphtheria is infectious so long as any bacilli remain in the fauces—a period which sometimes extends to from four to six, or even more weeks. In about half the cases, however, the bacilli ceased to be discoverable three days after the disappearance of the membrane.

One of the most interesting portions of the investigation had reference to the presence of the bacillus in the apparently unaffected members of families in which cases of diphtheria had arisen, for it was found that, varying apparently according to the degree of isolation practised, from 30 to 50 per cent. of the inhabitants of diphtheria-infected houses had the bacillus in their fauces, and that of these a considerable proportion ultimately developed the disease. Not all, however; and thus we have proof that people who go about and do not suffer may for two or three weeks carry these organisms within them, and so far as they are virulent be infectious to others.

Diphtheria is, like cholera and enteric fever, communicable solely by the discharges from the passages in which the bacilli grow, and if all people who harbour the bacilli can be isolated the spread of the disease should stop. Everything, however, depends on the early diagnosis of the cases and their continued isolation and disinfection until they are free from bacilli.

For both these purposes bacteriological examination is essential; with its aid a diagnosis can be arrived at in twelve hours, while without it the differentiation between the infectious and the harmless cases is difficult, if not impossible.

The Health Department of New York has accepted *in toto* the bacillary hypothesis, and has organised a very complete machinery for putting at the disposal of the medical profession the means necessary for obtaining a proper prompt bacteriological diagnosis in all cases in which diphtheria is suspected. Numerous depôts are established in various parts of the city where physicians can obtain, free of cost, the materials necessary for preparing bacterial cultures. These are put up in little boxes, each containing two glass tubes, one having within it the sterilised culture medium, the other a sterilised swab of cotton-wool mounted on a wire. The tubes are closed with sterilised cotton-wool plugs, and each box has with it directions how the materials are to be used. The swab is to be applied to the inflamed portions of the throat, then drawn over the surface of the culture medium, and then replaced in its own tube. Each tube is then to be plugged with its own stopper and returned to the box, which is then to be sent back to the depôt from which it was obtained. At four o'clock every afternoon the boxes are collected from all parts of the city, placed in an incubator, and by noon on the following day the diagnosis is ready to be sent to the physician, either by post or telephone. Cases which prove to be false diphtheria are not visited by the Health Department inspectors, but those in which the bacillus is found are subjected to the usual rules and regulations which apply to cases of contagious disease, among which is isolation until infection ceases; in other words, until the bacillus ceases to be discoverable.

The process of bacteriological diagnosis has now been in operation for more than a year, and has been found to be thoroughly reliable; even in cases of purely laryngeal diphtheria cultures can be obtained from the fauces, although not always by a single examination.

Working on the large scale, as is done in New York, the expense does not seem to be great. It appears that about 100,000 sets of culture materials have been made, and that they cost about six cents a box. Sometimes as many as 90 examinations are made in a day, but the daily average for six months had been 60.

The advantage of being able not only to diagnose in its earlier stages a disease which is often difficult of recognition, but to pick out from a family in whose midst a first case has occurred those members who have become tainted with the infection before any symptoms have arisen must be very great, and if the treatment by antitoxines, of which we hear so much from Berlin and other places, should prove to be reliable, may be of inestimable value, for this treatment is only available in the early stages of the disease. For the mere object, however, of enforcing early isolation, this method of diagnosis seems likely to be a matter of no small importance.

We should have liked further information as to whether the cases of so-called false diphtheria were ever followed by paralysis, the occurrence of which seems by many to be looked on as the touchstone, and we should also have been glad to know whether Dr. Biggs was prepared to maintain that no other throat disease except diphtheria was communicable from patient to patient. No doubt some things require further explanation, but even as it stands the communication of Dr. Hermann Biggs is a most important one, and the method now only awaits further testing by other sanitary authorities.

